# Response to: Comment on “Evaluation of Complication Rates after Breast Surgery Using Acellular Dermal Matrix: Median Follow-Up of Three Years”

**DOI:** 10.1155/2018/6917454

**Published:** 2018-08-01

**Authors:** Felix J. Paprottka, Nicco Krezdorn, Heiko Sorg, Sören Könneker, Stiliano Bontikous, Ian Robertson, Christopher L. Schlett, Nils-Kristian Dohse, Detlev Hebebrand

**Affiliations:** ^1^Department of Plastic, Aesthetic, Reconstructive and Hand Surgery, Agaplesion Diakonieklinikum Rotenburg, Elise-Averdieck-Straße 17, 27356 Rotenburg (Wümme), Germany; ^2^Harvard Medical School, Brigham and Women's Hospital, Department of Surgery, Division of Plastic Surgery, 75 Francis Street, Boston, MA 02115, USA; ^3^Department of Plastic, Reconstructive, Aesthetic and Hand Surgery, Alfried Krupp Krankenhaus, Hellweg 100, 45276 Essen, Germany; ^4^Department of Plastic, Aesthetic, Hand and Reconstructive Surgery, Hannover Medical School, Carl-Neubergstraße 1, 30625 Hannover, Germany; ^5^Department of Pathology, Agaplesion Diakonieklinikum Rotenburg, Elise-Averdieck-Straße 17, 27356 Rotenburg, Germany; ^6^Department of Surgery, Royal Brompton Hospital, Sydney St, London, UK; ^7^Department of Diagnostic and Interventional Radiology, University Hospital Heidelberg, Im Neuenheimer Feld 110, 69120 Heidelberg, Germany

We appreciate Dr. Heisterkamp's interest [1] in our article [2], which might be influenced by the payment he received from RTI-Surgical, the producer and distributor of Tutomesh, and we are happy to address his concerns.

Dr. Heisterkamp complained about our use of the term “ADM” for Tutomesh; in the scientific literature, Tutomesh is referred to by other authors as a bovine-derived ADM [3], but the title could be changed as follows: “Evaluation of Complication Rates after Breast Surgery Using Two Different Kinds of Acellular Dermal Matrix and One Bovine Pericardium Collagen Membrane: Median Follow-Up of Three Years.”

Furthermore, Dr. Heisterkamp raised concerns about scientific correctness when comparing different acellular membranes for breast reconstruction with respect to complication rates, but this approach is common scientific practice and therefore should not be considered a problem.

We admit that, in Tables 1, 2, and 4 and in the accompanying table legends, “median” values were mistakenly referred to as “average”. Furthermore, the term “Others” in Table 2 should be replaced by “Secondary augmentation after aesthetic-related complications”, which includes four incidents of IMF loss. Dr. Heisterkamp's assumption that 70% of Tutomesh patients (11 out of 16 patients) had an oncologic history is incorrect. The correct percentage of patients with an oncologic background in the BADM group is 44% (7 out of 16 patients) compared to, for example, the PADM cohort with 67% (14 out of 21 patients), as already stated in the legend of Table 2.

Our article was indeed based on a retrospective study design with a limited sample size; this potential bias was addressed throughout the manuscript. Also, we stated that “the inclusion of oncologic patients with breast reconstruction after/without radio- and/or chemotherapy may have resulted in greater heterogeneity and therefore increased complication rates”. Furthermore, we stated the following in the discussion: “Other limitations of our study include the relative brevity of follow-up with a median time period of 36 months. Many of the complications occurred early in the follow-up interval. Statements about the incidence of long-term complications such as capsular contractures are limited. Certainly, a larger patient collective and a longer follow-up interval are needed to obtain statistically significant results concerning complication rates.”

The concerns regarding the correctness of our statistical analyses, which were raised by Dr. Heisterkamp, are from our point of view not supported. First of all, two endpoints were prospectively defined: (1) the primary endpoint as a composite of skin necrosis, seroma, haematoma, infection, recurrence of capsular contracture, implant malposition, and implant loss; (2) the secondary endpoint included all complications such as the primary endpoint in addition to occurrence of Red Breast Syndrome (RBS). All the reanalysis in the commissioned letter addressed only the primary endpoint. In our article, no significant differences were revealed or reported regarding the primary endpoint. Thus, Dr. Heisterkamp's conclusion—that there is a substantial disagreement between his and our analysis—is not accurate; neither analysis shows a statistically significant difference.

However, our main findings were regarding the secondary endpoint. We showed that there was a statistically significant difference for BADM versus HADM regarding the secondary endpoint (44%  [7/16] versus 7%  [1/15], respectively) with a *p* value of 0.02 using the Chi-Square test. When instead using Fisher's Exact Test, as recommended by Dr. Heisterkamp, this reported difference remains statistically significant (*p* = 0.04). Thus, our findings are the same using either of these two statistical approaches.

Regarding the potential age differences in the populations, we believe that, due to the limited sample size, an overstretching of the statistics with ANOVA or addressing these differences in a multivariate analysis would not be appropriate.

Surely, a prospective randomized study would help to validate the presented results and should be our next step. Accordingly, the dedication of RTI-Surgical would be highly appreciated. We believe our work has value due to direct comparisons of different acellular membranes used for breast reconstruction/augmentation being rare in the literature. Daily clinical practice has confronted the treating surgeon with various complications regarding the use of ADMs. Moreover, it is not only one particular brand or product that is connected with these issues; it is the whole group of these materials. Therefore, ADMs and related products should be used with care and decision-making should be made individually for each patient case.
